# Ultrasound features of shoulder involvement in patients with ankylosing spondylitis: a case–control study

**DOI:** 10.1186/1471-2474-14-272

**Published:** 2013-09-22

**Authors:** Sanae Ali Ou Alla, Rachid Bahiri, Hanaa Amine, Hourya El Alaoui, Hanane Rkain, Souad Aktaou, Redouane Abouqal, Najia Hajjaj-Hassouni

**Affiliations:** 1 Department of Rheumatology, El Ayachi Hospital, University Hospital of Rabat-Sale, Rabat-Sale, Morocco; 2 Clinical Research and Epidemiology, Faculty of Medicine, Rabat, Morocco

**Keywords:** Ankylosing spondylitis, Shoulder, Ultrasound

## Abstract

**Background:**

During Ankylosing spondylitis (AS) courses, shoulder involvement is common. However, etiologies of shoulder pain in patients with AS remain to be defined. The aim of this study was to investigate the prevalence of ultrasound (US) abnormalities in shoulders of patients with ankylosing spondylitis (AS), and to determine predictive factors of ultrasound shoulder enthesitis.

**Methods:**

38 patients with AS were included with 38 age and sex-matched healthy controls. All patients fulfilled the modified New York criteria for ankylosing spondylitis. Clinical and demographical data were recorded. US examination of bilateral shoulders was performed by a musculoskeletal sonographer according to a defined protocol that included imaging of the insertions of supraspinatus, subscapularis and infraspinatus tendons, rotator cuff tendons, subacromial-subdeltoid bursa, acromioclavicular joint, and glenohumeral joint.

**Results:**

The mean age of patients and controls was 36 years, each group of patients and controls comprised 22 men (57.9%) and 16 women (42.1%). Disease duration was 9.6 ± 7.2 years. Among 38 patients with AS, 21 had coxitis (55%) and 19 had previous or current shoulder pain (50%). AS shoulders presented significantly more ultrasound enthesitis than controls shoulders (43 shoulders (56.6%) versus 8 shoulders (10.5%) respectively). Involvement of rotator cuff tendons was significantly higher in patients with AS compared with control subjects (16/38 (42.1%) versus 6 (15.2%) respectively). However, involvement of gleno-humeral and acromio-clavicular joints was infrequent in both groups. In patients with AS, we found that the presence of coxitis was the only significant predictive factors of shoulder enthesitis (Odds Ratio (OR) = 9.4; Confidence interval (CI) 95% (1.10; 81.9), p = 0.04).

**Conclusions:**

Ultrasound abnormalities of shoulders are common in patients with AS, and the most frequent abnormalitie was enthesitis, which was associated with the presence of coxitis.

## Background

During Ankylosing spondylitis (AS) courses, shoulder involvement is common [1,2]. On the basis on AS clinical assessment, prevalence of shoulder pain varied from 3.5 to 33% [1-4]. Despite its common occurrence in patients with AS, shoulder involvement is not frequently disabling [2]. However, etiologies of shoulder pain in patients with AS remain to be defined, physicians presumed that shoulder pain may be due to synovitis, bursitis, or structural joint damage. There have been very few studies which examined the etiology of shoulder pain in patients with AS, and usually not in a controlled format [1,2,4,5]. Some cross-sectional plain radiographic studies have described joint space narrowing, erosion, and bony proliferation in the acromio-clavilcular joint, the gleno-humeral joint, and around the rotator cuff insertion [1,2,5].

Nowadays, it is widely known that enthesitis is the primary clinical feature in AS [6,7]. Enthesitis has been demonstrated in various peripheral locations such as the Achilles tendon, and the knee [8-10], but it has not yet been formally evaluated in the shoulder. For the clinical evaluation of enthesitis in AS, several index have been developed [11-16], but the shoulder is not usually included in these index.

Over the last few years, ultrasound has proved to be a reliable method for assessing tendon and joint involvement with a high sensitivity. Several musculoskeletal features of AS such as enthesitis, synovitis, erosion, bursitis, and tenosynovitis could be visualized by ultrasound [17]. Recently, there is an increasing interest for the use of ultrasound for AS evaluation. Many ultrasound index were developed to assess enthesitis in patients with AS: the Glasgow Ultrasound Enthesitis Scoring System (GUESS) [18], Sonographic Entheseal Index (SEI) [19], and Ultrasound Enthesis Score [20]. None of these indices include the scanning of shoulder enthesis, which due probably to the lack of sonographic study of shoulder in patients with AS.

The purpose of this study was therefore to assess ultrasound features of shoulder in AS patients compared with age and sex-matched healthy controls, furthermore, we aim to determine predictive factors of shoulder enthesitis in patients with AS.

## Methods

Thirty eight patient with AS and 38 age and sex-matched healthy controls were enrolled. All patients with AS fulfilled the modified New York criteria for ankylosing spondylitis [21]. We collected demographic and clinical data including; disease duration, form of AS, presence of coxitis and previous or current shoulder pain.

AS activity was assessed by the bath ankylosing spondylitis disease activity index (BASDAI) [22], and we used the bath ankylosing spondylitis function index (BASFI) [23] to evaluate function. Each patient and control subject provided informed written consent to participate in this study at baseline and the study received approval from the ethic committee of biomedical research of Faculty of Medicine and Pharmacy of Rabat.

### Ultrasound

Bilateral US examination of shoulders, for each patient and control subject, was realized by a rheumatologist with 2 years of experience in musculoskeletal US, assisted by a rheumatologist with 7 years of experience in musculoskeletal US. Toshiba Xario equipment with a 14-MHz linear array transducer was used.

We realized standarized sections [24-29] to scan each tendon of cuff rotator in both longitudinal and transverse planes as following: 1) neutral position of shoulder, elbow flexed 90, scaning of biceps tendon between the greater and less tuberosities in ventral transverse and longitudinal planes for visualisation of the tendon and detection of minute fluid accumulations and detection of tenosynovitis, 2) during maximal external rotation with the elbow flexed 90 and fixed on the iliac crest, scaning of the subscapularis tendon with visualisation of its enthese on the lesser tuberosity 3) supraspinatus was evaluated along its long and short-axis while the patient was placing the arm posteriorly and the palmar side of the hand on the superior aspect of the iliac wing, its insertion on the greater tuberosity was examined 4) the patient placing the hand on the opposite shoulder, infraspinatus was scanned with its enthese on the greater tuberosity. Blood flow was examined at the entheseal sites using power Doppler mode with a pulse repetition frequency of 750 Hz and a power Doppler gain of 60 dB.

In B mode, we explored cuff rotator tendon morphology to detect tendinopathy by searching partial or full-thickness tears and intra-tendinous calcifications. Presence of tenosynovitis of the long biceps tendon was assumed when the echogenic tendon was surrounded by a hypoechogenic band on the transverse and longitudinal sections. At tendon insertion, we searched for the following abnormal findings, and any one of them was considered as a feature of enthesitis [30]; tendon thickening at the level of bony attachment, enthesophytes, bony erosion and presence of Doppler signal at the level of bony attachment. While assessing rotator cuff, subacromial or subdeltoid bursitis was searched.

Gleno-humeral and acromio-clavicular joints were also assessed as following: dorsal transverse section through the infraspinous fossa laterally below the scapular spine and axillary longitudinal section, for detection of synovitis, synovial proliferation, and erosion of the humeral head; and ventral transverse section over the acromio-clavicular joint.

### Statistical analysis

Continuous variables were expressed by median ± standard deviation or median (interquartile ranges), and categorical variables as number (percentage). Comparison of categorical variables between patients and control subjects were done by chi-square or Fisher’s exact test. Continuous variables were compared by t-student test. Furthermore, we used chi-square test to compare the frequency of enthesitis between subgroups in patient group, these subgroups were defined functions of AS subtypes.

Thereafter, we performed a logistic regression to determine predictive factors for enthesitis in AS patients; firstly univariate logistic regression were done, and the remaining factors (P < 0.05) were entered into a final global multivariate logistic regression model, so multivariate analysis were secondly performed.

Results were considered significant for p < 0.05, and Confidence intervals (CI) were computed at the 95% level. A computer software package (SPSS, version 13; SPSS, Chicago, IL) was used to perform all statistical calculations.

## Results

Each group of patients with AS and control subjects comprised similarly 22 men (57.9%) and 16 women (42.1%), the mean age was respectively 36.8 ± 10.7 and 36.9 ± 10.6 years (p = 0.95). AS patients had mean disease duration of 9.6 ± 7.2 years. The mean BASDAI was 4.9 ± 1.9. Among 38 patients with AS, 21 had coxitis (55%) and 19 had previous or current shoulder pain (50%) (Table 1).

**Table 1:** Comparison of characteristics and ultrasound results between AS patients and control subjects

	**Patients**	**Control subjects**	**p**
	**(n = 38)**	**(n = 38)**	
**Age (years)**	36.8 ± 10.7	36.9 ± 10,6	0.95
**Sex: males**	22 (57.9)	22 (57.9)	1
**Disease duration (years)**	9.6 ± 7.2		
**Previous treatment**			
NSAIDs	26 (68.4)		
Methotrexate	3 (7.9)		
Sulfasalazine	8 (21.1)		
Anti-TNF drugs	1 (2.6)		
**Current treatment**			
NSAIDs	22 (57.9)		
Methotrexate	1 (2.6)		
Sulfasalazine	2 (5.3)		
Anti-TNF drugs	13 (34.2)		
**AS subtypes**			
Isolated axial disease	10 (26.3%)		
Axial and enthesic	9 (23.7%)		
Axial with peripheral	5 (13.2)		
Axial and enthesic and peripheral	14 (36.8)		
**Pain of shoulder**	19 (50)		
**coxitis**	21 (55.3)		
**BASDAI**	4.9 ± 1.9		
**BASFI**	5.1 ± 2.9		
**Enthese involvement**	26 (68.4)	8 (21.1)	<0.001
Supraspinatus	22 (57.9)	5 (13,2)	<0.001
Subscapularis	14 (36.8)	1 (2.6)	<0.001
Infraspinatus	15 (39.5)	3 (7.9)	0.001
Multiple enthesitis	14 (36.8)	0 (0)	<0.001
Bilateral enthesitis	17 (44.7)	0 (0)	0.003*
**Rotator cuff tendinopathy**	16 (42.1)	6 (15.8)	0.01
Supraspinatus	11 (28.9)	3 (7.9)	0.01
Subscapularis	5 (13.2)	1 (2.6)	0.20*
Infraspinatus	8 (21.1)	3 (7.9)	0.10
Biceps tenosynovitis	5 (13.2)	0 (0)	0.05*
Bilateral involvement	12 (31.5)	1 (2.6)	0.02*
**Gleno-humeral joint**			
Synovitis	1 (2.6)	0 (0)	1*
erosion	1 (2.6)	0 (0)	1*
**Acromio-claviculaire joint**			
Erosion	0 (0)	3 (7.9)	0.24*
synovitis	0 (0)	0 (0)	
**subacromial bursitis**	0 (0)	0 (0)	

*exact Fisher’s test.

Results are expressed by mean ± standard deviation or number (percentage).

**Figure 1:**
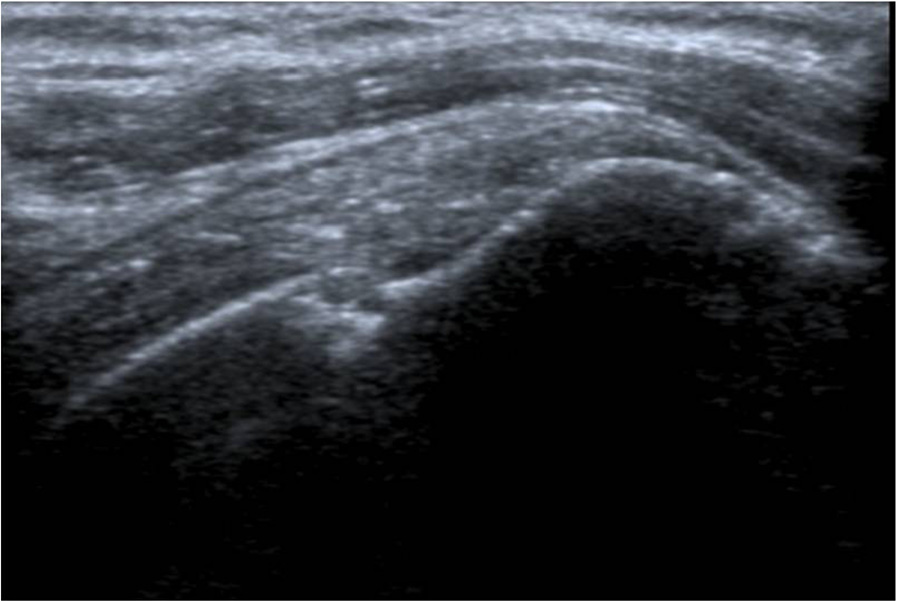
Ultrasound image of supraspinatus enthesitis: sagittal B-mode US scanning of supraspinatus tendon showing bony erosion with enthesophytes.

**Figure 2:**
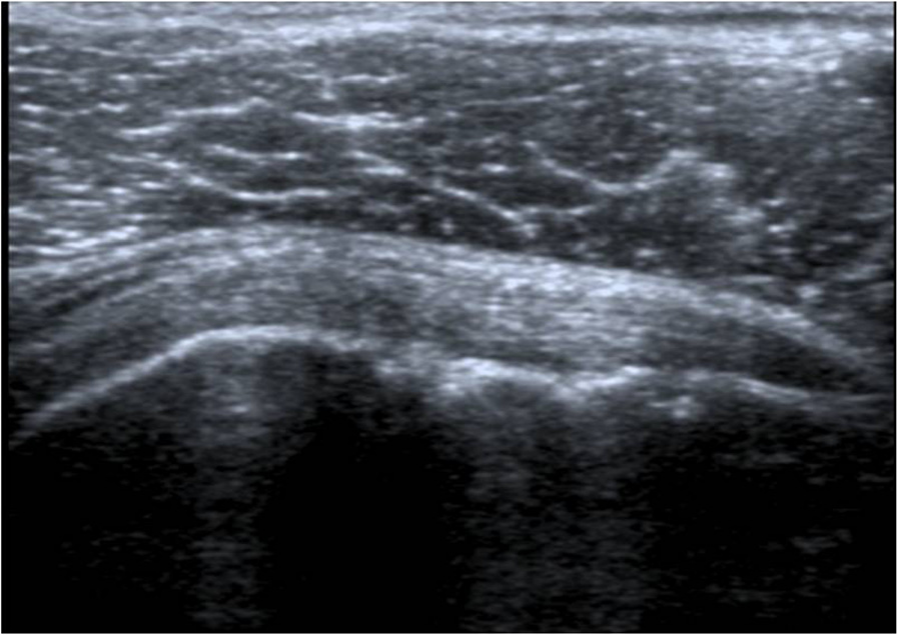
Ultrasound image of subscapularis enthesitis: sagittal B-mode US scaning of subscapularis tendon showing bony erosion with enthesophytes.

Ultrasound abnormalities of shoulders were significantly more frequent in patients with AS (28/38 patients (73.7%)) than controls (14/38 controls (36.8%)). The most frequent disorder in patients with AS was enthesitis. Among 76 shoulders examined in each group (Table 2), 43 AS shoulders presented enthesitis (56.6%) versus 8 shoulders in control group (10.5%). In both groups, the most frequent involved entheses in shoulder were supraspinatus e.g. (Figure 1) followed by subscapularis e.g. (Figure 2) and infraspinatus. Table 3 shows aspects of enthesitis in AS shoulders compared with control shoulders.

**Table 2:** Comparison of ultrasound abnormalities between AS shoulders and control shoulders

	**AS shoulders**	**Control shoulders**	**p**
	**(n = 76)**	**(n = 76)**	
**Enthese involvement**	43 (56.6)	8 (10.5)	<0,001
Supraspinatus	31 (40.8)	5 (6.6)	<0.001
Subscapularis	21 (27.6)	1 (1.3)	<0.001
Infraspinatus	22 (28.9)	2 (2.6)	<0.001
Multiple enthesitis	21 (27.6)	0 (0)	0.01*
**Rotator cuff tendinopathy**	28 (36.8)	7 (9.2)	<0.001
Supraspinatus	16 (21.1)	4 (5.3)	0.004
Subscapularis	9 (11.8)	2 (2.6)	0.03*
Infraspinatus	12 (15.8)	3 (3.9)	0.01*
Biceps tenosynovitis	8 (10.5)	0 (0)	0.006*
Bilateral involvement	12 (15.8)	2 (2.6)	0.67*
**Gleno-humeral joint**			
Synovitis	1 (1.3)	0 (0)	1.00*
erosion	2 (2.6)	0 (0)	0.50*
**Acromio-claviculaire joint**			
Synovitis	0 (0)	0 (0)	0.24*
Erosion	0 (0)	3 (3.9)	
**Sub-deltoid bursitis**	0 (0)	0 (0)	

*exact Fisher’s test.

Results are expressed by number (percentage).

**Table 3:** Comparison of enthesitis aspects and rotator cuff tendon disorders between patient and control shoulders

	**AS shoulders**	**Control shoulders**	**P**
	**(n = 76)**	**(n = 76)**	
**Supraspinatus enthesitis**			
Doppler	1 (1.3%)	0 (0)	1
Enthesophytosis	4 (5.3%)	0 (0)	0.12
Bony erosion	30 (39.5%)	5 (6.6)	<0.001
Thickening	2 (2.6%)	0 (0)	0.49
**Subscapularis enthesitis**			
Doppler	0(0)	0(0)	1
Enthesophytosis	1 (1.3%)	0 (0)	<0.001
Bony erosion	22 (28.9%)	2 (2.6%)	1
Thickening	1 (1.3%)	0 (0)	
**Infraspinatus enthesitis**			
Doppler	0(0)	0 (0)	1
Enthesophytosis	1 (1.3%)	0 (0)	<0.001
Bony erosion	21 (27.6%)	1 (1.3%)	
Thickening	0 (0)	0 (0)	
**Supraspinatus tendon**			
Partial tear	10 (13.2%)	0 (0)	0.001
Full tear	2 (2.6%)	0 (0)	0.49
Calcification	4 (5.3%)	4 (5.3%)	1
Doppler	0 (0)	0 (0)	
**Infraspinatus tendon**			
Partial tear	6 (7.9%)	0 (0)	0.028
Full tear	1 (1.3)	0 (0)	1
Calcifications	2 (2.6%)	2 (2.6%)	1
Doppler	0 (0)	0 (0)	
**Subscapularis tendon**			
Partial tear	8 (10.5%)	0 (0)	0.006*
Full tear	1 (1.3%)	0 (0)	1
Calcifications	3 (3.9%)	3 (3.9%)	1
Doppler	0 (0)	0 (0)	

*exact Fisher’s test.

Results are expressed by number (percentage).

In addition, entheseal involvement was multiple in 21 of 76 AS shoulders and in 14 patients with AS (36.8%), however, there was not multiple entheseal involvement in control shoulders. Seventeen patients from 38 had bilateral enthesitis in shoulders (44.7%) while no bilateral enthesitis was found in control subjects.

Involvement of rotator cuff tendons was significantly higher in patients with AS compared with control subjects (16/38 (42.1%) versus 6 (15.2%) respectively). This rotator cuff involvement was significantly frequently bilateral in patients with AS in comparison with control subjects (Table 2). Description of rotator cuff tendon abnormalities is presented in Table 3. Other ultrasound abnormalities such as synovitis and joint involvement were similarly less frequents in both groups (Tables 2 and 3).

In the group of patients with AS, 23/38 patients had previously known entheseal involvement. Patients with previously known enthesitic form presented similar frequency of shoulder enthesis than patients without enthesitic form (16/23 (69.5%) and 10/15 (66.6%) respectively, p = 1). Similarly, no difference was found between patients with isolated axial disease and mixed forms (axial disease with peripheral joint involvement and/or enthesitic form).

Among 76 AS shoulders, thirty three shoulders were currently or previously painful. These painful shoulders presented significantly more ultrasound abnormalities (75.8% (25/33)) and more enthesitis (24/33 (72.7%)) compared with no painful AS shoulders (20/43 shoulders (46.5%) presented US abnormalities and 19/43 (44.2%) enthesitis).

At univariate analysis, the presence of shoulder enthesitis was associated to long onset disease, coxitis, high disease activity (BASDAI), and poor function (high BASFI). After multivariate analysis, the only factor which still strongly associated to shoulder enthesitis was coxitis (OR = 9.4 with IC 95% (1.10; 81.9)) (Table 4).

**Table 4:** Predictive factors of shoulder enthesitis in AS patients

	**Univariate analysis**	**Multivariate analysis**
	**OR (IC 95%)**	**OR (CI 95%)**
**Age (years)**	1.03 (0.96; 1.10)	
**Sex**	0.62 (0.15; 2.48)	
**Shoulder pain**	2.7 (0.65; 11.4)	
**AS subtypes**	1.13 (0.64; 2.00)	
**Disease duration (years)**	1.21 (1.02; 1.44)^a^	1.24 (0.96; 1.62)
**coxitis**	13,5 (2.36; 77.9)^b^	9.4 (1.10; 81.9)^e^
**BASDAI**	1.72 (1.10; 2.72)^c^	1.83 (0.84; 3.98)
**BASFI**	1.50 (1.10; 2.05)^d^	1.03 (0.65; 1.63)

^abcde^significant results. ^a^p = 0.02, ^b^p = 0.003, ^c^p = 0.02, ^d^p = 0.01, ^e^p = 0.04.

*OR* odds ratio, *CI* confidence interval.

BASDAI: the bath ankylosing spondylitis disease activity index.

BASFI: the bath ankylosing spondylitis function index.

## Discussion

To our knowledge, this work represents the first controlled ultrasound evaluation of shoulders in patient with AS. There were several important findings in this study: Firstly, gleno-humeral synovitis was uncommon in our patients with AS. Secondly, the high frequency of enthesitis in AS shoulders with multiple entheseal involvement in shoulder compared with controls, and the bilateral aspect of enthesitis in AS shoulders. Thirdly, the frequency of enthesitis in shoulders was similar in all AS subtypes. Fourthly, coxitis was the only predictive factor for this enthesitis.

Despite of the commonly involvement of shoulders during AS course [2], very few studies focused to study the etiology of shoulder pain in these patients [1,2,4,5]. Using plain radiography, shoulders of 26 AS patients were examined in a previous not controlled study [2]. 31% of patients presented evident radiologic abnormalities and the most commonly involved site was the acromio-clavicular joint, 9 shoulders presented sever radiologic changes in the gleno-humeral joint. One other study, which enrolled 52 patients with AS, found in 29 shoulders plain radiologic abnormalities including acromio-clavicular joint narrowing, sclerosis of the greater tuberosity and cystic changes in the humeral head [1]. In our study, ultrasound involvement of acromio-clavicular and gleno-humeral joints was rare. Recently, a MRI study included 15 patients with AS with shoulder pain, and a control group of 92 individuals with nonspecific shoulder pain. Bone marrow edema at any entheseal site was noted in significantly more AS shoulders (70.6%) than in control (19.1%) shoulders [4].

Some clinical scores have been developed to assess enthesitis in patients with AS, but they don’t all include the shoulder [11-16]. The most recent index has been developed by the Spondyloarthritis Research Consortium of Canada (SPARCC), it evaluates only the peripheral entheses and it includes the shoulder [16]. According to this SPARCC index, the most frequently affected sites were the greater trochanter and supraspinatus insertion. Despite the lack of specificity of enthese clinical evaluation, the finding of this index is relatively consistent with our ultrasound results, supraspinatus insertion was also the most involved enthese site in the shoulders of our patients.

For enthese evaluation, the accuracy of clinical examination and X ray is still uncertain. So, new imaging techniques such as ultrasound and magnetic resonance imaging (MRI) were recently used to examine enthese in patients with AS. Over the last few years, several studies have highlighted the value of ultrasound in assessing entheses in AS. Ultrasound B-mode aspects of lower limb enthesitis of AS was firstly described by Lehtinen et al. [10] in 1994, and in 2003 Balint et al. developped a ultrasound scoring system designed to assess five entheseal sites in the lower limb (GUESS) [18]. Thereafter, D’Agostino et al. used B-mode US combined with power Doppler (PDUS) to show a high frequency of abnormal peripheral enthesitis among patients with AS in comparison with controls affected by rheumatoid arthritis or degenerative spinal disease in a cross-sectional study [31]. Recently, other ultrasound scoring system were developed, the Spanish enthesis index [19,20] which is based on the use of ultrasound B-mode only, and the Madrid sonographic enthesitis index (MASEI) [20], which combines abnormalities in grey scale and Doppler . However, none of these scores included the shoulder, and actually there is no clear agreement on which structures to examine while assessing enthesitis in patients with AS. Also, there is no consensus of the definition of enthesitis in AS, so definition varied between authors [30]. In 2005, an OMERACT-EULAR working group on Ultrasound was constituted to address validity issues, and to establish international consensus and scoring systems in the use of US in musculoskeletal diseases [32].

On the other hand, the landmark of US enthesitis in patients with AS was the presence of abnormal vascularization at entheses [31]. In our work, abnormal vascularization at entheses in shoulder was rarely found, that could be due to the recruitment based on patients with or without current or previous shoulder pain. In addition, the high frequency of asymptomatic enthesitis is in agreement with previous findings in other enthese sites in patients with AS [10,18,31]. It is known that peripheral enthesitis may be observed in all forms of AS and all phases of disease evolution [33], also D’agostino et al. found no major differences in the frequency of Ultrasound enthesitis between the different AS subtypes [31], which is consistent with our findings.

In our work, synovitis of gleno-humeral joint was rare. On the other hand, patients with AS presented a high prevalence of coxitis in our study. Previous studies, which aimed to describe clinical aspects of AS in our area, had also found a high prevalence of coxitis [34-36]. In the present paper, hip involvement was the only significant factor associated with enthesitis in shoulders. These results seem to be near to previous data suggesting that involvement of the hip joint in AS is higher in patients with shoulder involvement. Although, disease duration was not a significant factor of shoulder involvement in our study, which is in contrast with previous finding which suggested that disease duration could predict shoulder involvement [2].

## Conclusions

In conclusion, in patients with established AS, ultrasound abnormalities of shoulder are common, particularly enthesitis. The significant association between coxitis and shoulder enthesitis led us to suggest that ultrasound evaluation of shoulder should be performed in patients with AS with hip involvement. Finally, there is a great interest to evaluate the diagnostic value of shoulder enthesitis by performing large studies in early onset AS.

## Competing interests

The authors declare that they have no competing interests

## Authors’ contributions

We declare that we participated in the study as following:

SAL, RB, HA and N H conceived the study and supervised its design, execution, and drafted the manuscript. SAL and RA did data management and statistical analysis. HR, SAK, HE participated in the design of the study and coordination and helped to draft the manuscript. All authors read and approved the final manuscript.

## Pre-publication history

The pre-publication history for this paper can be accessed here:

http://www.biomedcentral.com/1471-2474/14/272/prepub
